# Rumen biohydrogenation of linoleic and linolenic acids is reduced when esterified to phospholipids or steroids

**DOI:** 10.1002/fsn3.1252

**Published:** 2019-11-06

**Authors:** Saman Lashkari, Majbritt Bonefeld Petersen, Søren Krogh Jensen

**Affiliations:** ^1^ Department of Animal Science AU Foulum Aarhus University Tjele Denmark; ^2^ Graasten Landbrugsskole Graasten Denmark

**Keywords:** biohydrogenation, cholesterol esters, phospholipids, triglycerides, unsaturated fatty acids

## Abstract

Manipulation of rumen biohydrogenation (BH) is of great importance, since decreased BH of linolenic acid (LNA) and linoleic acid (LA) is linked to increased content of the beneficial polyunsaturated fatty acids (PUFA) in dairy products and decreased content of trans fatty acids (FAs). We hypothesized that PUFA esterified to the complex lipid fractions are less prone to BH compared with PUFA esterified to the simple lipid fractions due to reduced lipolysis. In vitro rumen BH of different single lipid fractions was investigated, including free fatty acids (FFA), and esterified FA to triglycerides (TG), cholesterol esters (CE), and phospholipids (PL). A mixture of a buffer solution and rumen fluid was incubated with different lipid fractions, and C18 FAs were quantified by gas chromatography. In vitro BH kinetic parameters were quantified according to Michaelis–Menten equation and the maximum BH (V_max_) and time to achieve 50% of maximum amount (K_M_) estimated. Regardless of fatty acids, BH in CE and PL was lower than FFA and TG. The highest amount of cis‐9, trans‐11 conjugated linoleic acid (CLA) and trans‐10, cis‐12 CLA was observed in lipid fractions containing LA and LNA, respectively, regardless of lipid fractions. The present study demonstrates the importance of lipid fractions on BH of LNA and LA and formation of CLA isomers. The results show that BH of FAs depends on the lipid fractions.

## Graphical abstract

Linolenic and linoleic acids esterified to the complex lipid fractions are less prone to biohydrogenation compared with linolenic and linoleic acids esterified to the simple lipid fractions due to reduced lipolysis, which ultimately results in decreased biohydrogenation.

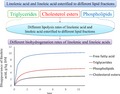

## 1 INTRODUCTION

Milk fat composition of cow and goat is composed of more than 400 different fatty acids (FAs) as a consequence of the high microbial activity in the rumen and the de novo synthesis in the mammary gland (Chilliard et al., 2007). The major FAs in bovine milk are typically C14:0 (10%–12%), C16:0 (25%–30%), C18:0 (8%–10%), and C18:1 (20%–30%), while PUFA normally account for <2% of the FA (Jensen, 2002). Similarly, beef and lamb meat contain less than 4 and 5.7% PUFA, respectively (Wood et al., 2004). Manipulating of FA composition of meat and dairy products to increase the content of nutritional beneficial PUFA and decrease the content of unhealthy trans FA has attracted great interest over several decades (Chilliard et al., 2007; Lashkari, Azizi, & Jahani‐Azizabadi, 2015; Lourenço, Van Ranst, Vlaeminck, De Smet, & Fievez, 2008; Palmquist, Beaulieu, & Barbano, 1993).

Dietary PUFA undergo the greatest changes during their passage from feed to dairy products due to the extensive BH taking place in the rumen (Chilliard et al., 2007). The mechanism facilitating and controlling rumen BH is not well known (Buccioni, Decandia, Minieri, Molle, & Cabiddu, 2012). The available free carboxylic group is a prerequisite for BH, and therefore, the lipolysis of lipid complexes is a prerequisite step prior to BH (Dawson & Hemington, 1974). Earlier studies have shown that although lipolysis takes place in the herbage itself, the rumen microorganisms are the main responsible for lipolysis in the ingested plant lipid complexes (Hespell & O'Bryan‐Shah, 1988). The number of microorganisms capable of hydrolyzing esters in the cultivated rumen microorganisms is low (Buccioni et al., 2012; Enjalbert, Combes, Zened, & Meynadier, 2017) and is mainly associated with *Buturivirio fibrisolvens* and *Anaerovibrio lipolytica* strains, and the strains are highly specialized (Buccioni et al., 2012; Hespell & O'Bryan‐Shah, 1988). Early research on lipase activities in the rumen has primarily focused on the lipase activity in galactolipids (Dawson & Hemington, 1974) and PL (Dawson, Hemington, Grime, Lander, & Kemp, 1974), which are the major lipid complexes present in grass and legume (Buccioni et al., 2012; Dawson et al., 1974). Researches indicate that glycolipids and PL are hydrolyzed as rapid as TG in in vitro experiment (Dawson, 1959; Dawson & Hemington, 1974). Petersen and Jensen (2014) have previously shown that in vitro BH differs between plant species. Studies on *B. fibrisolvens* esterase activity in rumen fluid have shown that enzyme activity declines with increasing chain length of the esterified FA (Hespell & O'Bryan‐Shah, 1988; Lanz & Williams, 1973). Tocopherol esters seem not to be hydrolyzed in the rumen of dairy cows (Hymøller & Jensen, 2010) and the same is hypothesized for more complex FA esters such as sterol esters. It is well established that only a small proportion of PUFA from vegetable oil rich in triglycerides are transferred to milk when fed to dairy cattle, but a much larger proportion of PUFA is transferred to milk when different herbage species are fed (Buccioni et al., 2012; Cabiddu et al., 2010; Hauswirth, Scheeder, & Beer, 2004; Petersen & Jensen, 2014; Petersen, Søegaard, & Jensen, 2011). However, the exact mechanism behind the different responses to BH in different herbage species has not been elucidated and quantified. Therefore, we hypothesized the BH of PUFA is responsive to lipid fractions, and PUFA esterified to the complex lipid fractions are less exposed to BH. The aim of the present study was to investigate in vitro rumen BH of LNA and LA, BH end product, and formation of CLA isomers from LNA and LA esterified to TG, PL, or CE.

## 2 MATERIALS AND METHODS

### 2.1 Treatment of donor cows

Three lactating Holstein cows (138 ± 19 days in milk, daily milk yield: 30 ± 4 kg), fitted with ruminal cannulas, housed in the experimental barn at the Aarhus University were used. The cows were fed twice per day with a grass‐based TMR diets (3.2% crude fat, 16% crude protein, and 34% NDF on DM basis) at 09.00 a.m. and 18.30 p.m. Cows were milked at 08:00 a.m. and 17:45 p.m.

### 2.2 Preparation of rumen fluid

Rumen fluid for the BH experiment was collected before morning feeding and milking, filtered through double‐layered cheesecloth, and then immediately transferred in sealed and preheated vacuum flasks under anaerobic conditions to the laboratory.

### 2.3 Preparation of test lipid fractions

Six different lipid fractions were studied, including FFA (linolenic; Sigma‐Aldrich, L2376, and linoleic acid; Sigma‐Aldrich, 62240), TG (glyceryl trilinolenate ≥97% and glyceryl trilinoleate ≥98%; Sigma‐Aldrich, T7140), CE (cholesteryl linoleate ≥98%; Sigma‐Aldrich, C0289), and PL (Lecithin Granulat, biosym.dk, 16637). The PL contained both LA and LNA.

Due to a very low FA content (9.9 g/kg), straw was chosen as carrier for the lipid fractions used for the BH experiment. The lipid fractions were solubilized in 100 ml diethyl ether and mixed with finely ground wheat straw (particle size: <1 mm). The mixture was prepared for all samples at once, and each incubated sample contained either 5 mg FFA, 10 mg TG or CE, or 15 mg of PL. After mixing, these mixtures were placed in a fume cupboard, and the added ether was allowed to evaporate, while the mixture was frequently stirred to ensure homogeneous samples. Mixtures containing lipid fractions with LNA (FFA and TG) were placed under a stream of N_2_ to prevent oxidation of the LNA.

### 2.4 In vitro incubation

In vitro incubation was performed according to Lashkari, Hymøller, and Jensen (2017). 500 mg of the prepared lipid–straw mixture, 22 ml of strained rumen fluid, and 22 ml of buffer solution were transferred to 60‐ml tubes. The buffer solution was prepared according to McDougall (1948). The lid of the tube was punctured with an injection needle and then equipped with a syringe to allow gas to escape from the tubes without compromising the anaerobic environment. Incubation was carried out in a water bath at 38°C and stopped at 0, 0.5, 1, 2, 4, 6, and 11 hr after incubation by transferring the test tubes into ice slurry. Throughout the experiment, pH was monitored in the samples. The contents of the tubes were freeze‐dried and stored at −20°C until further analysis.

### 2.5 Fatty acid analysis

Lipids were extracted in a mixture of chloroform and methanol (Bligh & Dyer, 1959) after acidification by boiling in 3 mol/L of HCl for 1 hr and converted into methyl esters as described by Jensen (2008) and quantified according to Jensen and Nielsen (1996) with small adjustments to the temperature program. Briefly, 250 mg of the freeze‐dried samples were weighed into culture tubes and extracted with 3 ml chloroform, 4 ml methanol, 1.5 ml distilled water, and 5 mg of internal standard C17:0 (heptadecanoic acid; Sigma‐Aldrich). The extracts were centrifuged for 10 min at 2,000*g*. After extraction, 1 ml of each extract was transferred to a new culture tube, evaporated to dryness under a stream of N_2,_ and methylated. Fatty acids were methylated with NaOH in methanol (0.5 M), sealed with Ar, and placed at 100°C for 15 min. After cooling, 1 ml of boron trifluoride was added, and tubes were sealed with Ar and placed at 100°C for 45 min. Fatty acid methyl esters (FAMEs) were finally extracted with 1 ml of heptane and analyzed with a gas chromatograph (Hewlett Packard 6890 Series; Agilent Technologies) equipped with an automatic column injector (Hewlett Packard 7673), a capillary column of 30 m × 0.32 mm inner diameter, 0.25 µm thickness (Omegawax™ 320; Supelco, Sigma‐Aldrich), and a flame‐ionization detector. The initial temperature was set to 170°C, and then, the temperature was raised to 200°C at the rate 2°C/min. The 200°C was held for 5 min and finally raised to 220°C at the rate 5°C/min. Fatty acids were identified by comparison of retention times with external standards (GLC‐68C; NU‐PREP‐CHECK). Up to five unidentified peaks eluted between the peaks of C18:1n‐9 and LA. These peaks were most likely different C18:1 isomers, but the peaks were not further identified.

### 2.6 In vitro biohydrogenation calculation

Based on the obtained data, the Michaelis–Menten equation was chosen as the most appropriate model to estimate the BH kinetic parameters**.** In vitro rumen BH of LNA and LA and formation of stearic acid (SA) fitted well to the Michaelis–Menten equation:

$$V=V_{\max}\times \frac{\left[s\right]}{k_{M}+\left[s\right]}$$

The obtained data in the in vitro BH experiment were analyzed by linear regression according to the Hanes plot as the time (hr) against the time divided with the amount of LNA or LA disappeared or SA formed (hr g^−1^ kg^−1^ DM). The V_max_ (g FA/kg DM) was calculated as the reciprocal slope, and K_M_ (hr) was calculated as the intercept multiplied with V_max_. V_max_ is asymptotic maximum amount of LA and LNA BH or maximum amount of SA formation, and K_M_ is the time (in hr) need to achieve 50% of asymptotic maximum LA and LNA BH or SA formation. For LNA and LA, their initial concentrations were subtracted from the concentration at the time when incubation was stopped (e.g., time = 0 hr subtracted from time = 0.5, 1, 2, 4, 6, and 11 hr) giving the total sum of products from the BH at the given time.

### 2.7 Statistical methods

Statistical analysis was performed with the PROC MIXED procedures in SAS package, ver. 9.2, with lipid fractions as fixed effects. In order to determine the effect of donor cows, three cows made up the triplicates per treatment. The effect of cow was tested, but no significant effect was found, and cow was included as a random effect in the final model. Differences in mean treatment effects were tested using the PDIFF command. The general model was as follows:

$$Y_{\mathrm{ij}}=\mu +LF_{i}+C_{j}+e_{\mathrm{ij}}$$

where *µ* is the overall mean, *LF_i_* is fixed effect of lipid fractions, *C_j_* is the random effect of cow, and *e_ij_* is the residual error. Random effects were assumed to be normally distributed with mean value 0 and constant variance, *C_j_* ~ *N* (0, $\sigma_{C}^{2}$) and *e_ij_* ~ *N* (0, *σ* ^2^). Significance was accepted at *p* ≤ .05 and a tendency .05 ≤ *p* ≤ .10.

## 3 RESULTS

### 3.1 Mixed lipid, straw, and rumen fluid fatty acid composition

The FA content of fine grinded straw, rumen fluid, and the individual lipid fractions mixed with straw and rumen fluid is shown in Table 1. Comparable amounts of LA and LNA were achieved in the FFA and TG samples, and the lowest concentration was observed in the PL and CE samples. The contribution of straw in total FA of the incubation tube was approximately 50%. However, only 7%–8% of LNA in FFA_LNA and TG_LNA, 17%–19% of LA in FFA_LA and TG_LA, and 50% of PL_LA and CE_LA were supplied by straw.

**Table 1:** Total fatty acid content (g/kg DM) and fatty acid composition (g FA/100 g FA) of straw, rumen fluid, and initial fatty acid content of in vitro samples (0 hr, average ± *SEM*; *n* = 3)

	Total fatty acid content	Myristic acid	Palmitic acid	Stearic acid	Oleic acid	Trans‐vaccenic acid	Linoleic acid	Linolenic acid	Cis−9, trans−11 CLA	Trans−10, cis−12 CLA
Straw	9.9 ± 0.4	5.67 ± 0.4	31.6 ± 0.3	6.2 ± 1.0	10 ± 0.3	0	29.1 ± 0.7	11.6 ± 0.4	0	0
Rumen fluid^†^	70.5 ± 3.4	1.27 ± 0.08	26.7 ± 1.98	52.9 ± 2.51	4 ± 0.40	5.35 ± 0.50	6.06 ± 0.68	1.74 ± 0.07	0.04 ± 0.009	0.03 ± 0.004
Fatty acid in culture tube at zero hour (lipid fraction + straw +rumen fluid)										
FFA_LNA	59.2 ± 3.1	1.33 ± 0.07	20.8 ± 0.5	36.3 ± 2.1	3.4 ± 0.3	3.6 ± 0.3	5.9 ± 0.5	26.4 ± 1.35	0.05 ± 0.01	0.03 ± 0.002
TG_LNA	58.8 ± 1.9	1.25 ± 0.02	20.1 ± 0.2	35.6 ± 2.1	3.4 ± 0.2	3.5 ± 0.3	6.0 ± 0.5	28.1 ± 1.13	0.09 ± 0.03	0.03 ± 0.009
FFA_LA	61.3 ± 4.2	1.23 ± 0.02	19.1 ± 0.6	33.2 ± 1.2	3.3 ± 0.2	3.4 ± 0.3	34.6 ± 0.1	1.9 ± 0.05	1.04 ± 0.21	0.06 ± 0.01
TG_ LA	47.5 ± 1.1	1.22 ± 0.05	18.0 ± 0.9	30.0 ± 1.2	3.3 ± 0.2	3.0 ± 0.3	40.2 ± 2.5	2.2 ± 0.02	0.48 ± 0.10	0.05 ± 0.007
CE_ LA	39.6 ± 1.3	1.47 ± 0.05	21.5 ± 0.5	35.3 ± 0.6	4.0 ± 0.1	3.6 ± 0.3	29.2 ± 1.2	2.5 ± 0.01	0.23 ± 0.03	0.03 ± 0.006
PL^§^	43.0 ± 1.7	1.38 ± 0.04	26.2 ± 0.4	39.4 ± 1.4	6.0 ± 0.2	4.2 ± 0.1	17.4 ± 1.0	3.0 ± 0.11	0.32 ± 0.06	0.03 ± 0.01

Abbreviations: CEs, cholesterol esters; CLA, conjugated linoleic acid; FFA, free fatty acid; LA, linoleic acid; LNA, linolenic acid; PL, phospholipids; TG, triglycerides

^†^ Rumen fluid contained 2.33% DM.

^§^ Phospholipids contain both linoleic and linolenic acids.

### 3.2 Biohydrogenation of linolenic acid and linoleic acid

**Figure 1:**
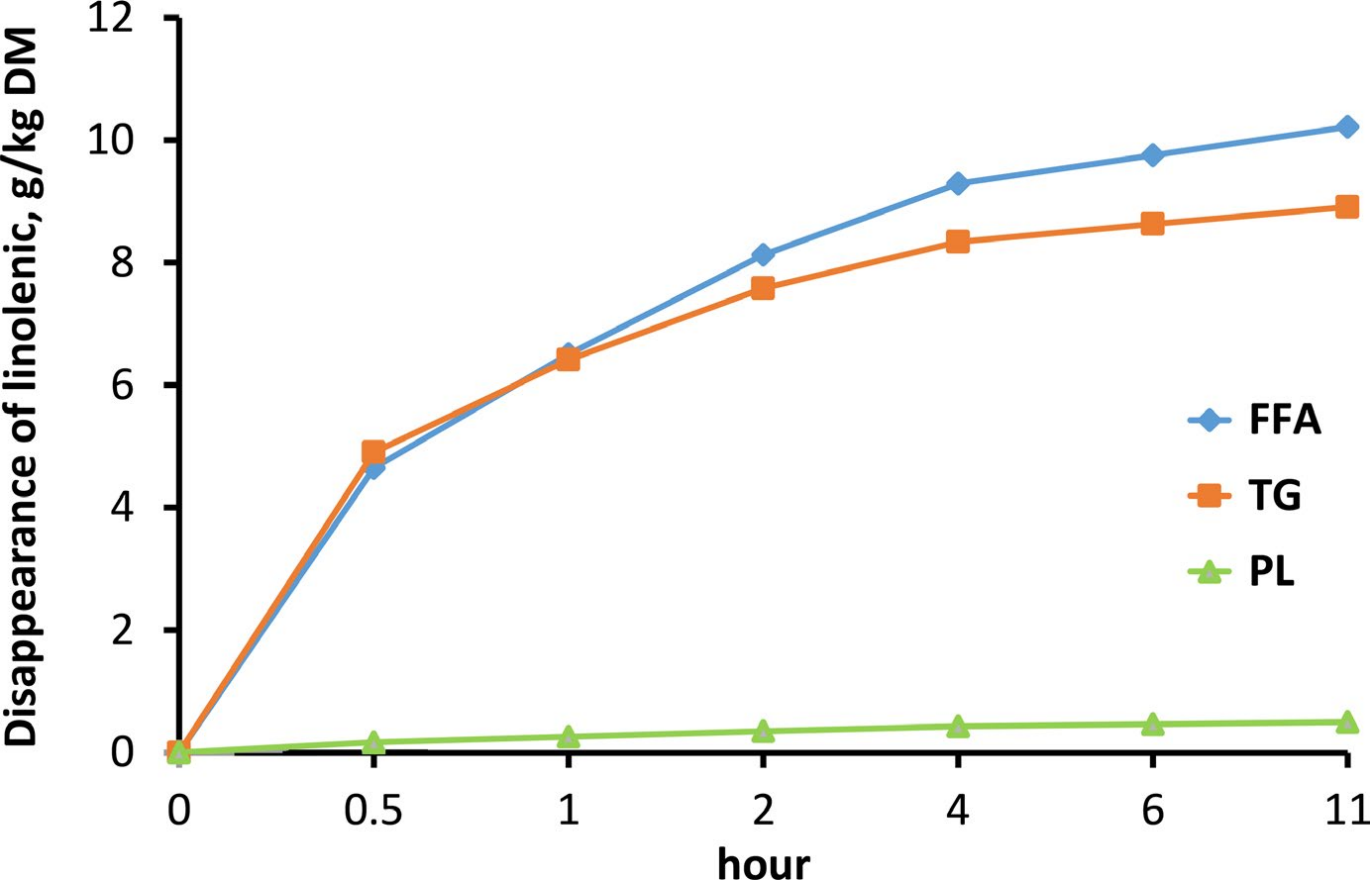
Disappearance of linolenic acid as free fatty acid (FFA), esterified to phospholipids (PL) or triglycerides (TG) modeled according to Michaelis–Menten equation

**Figure 2:**
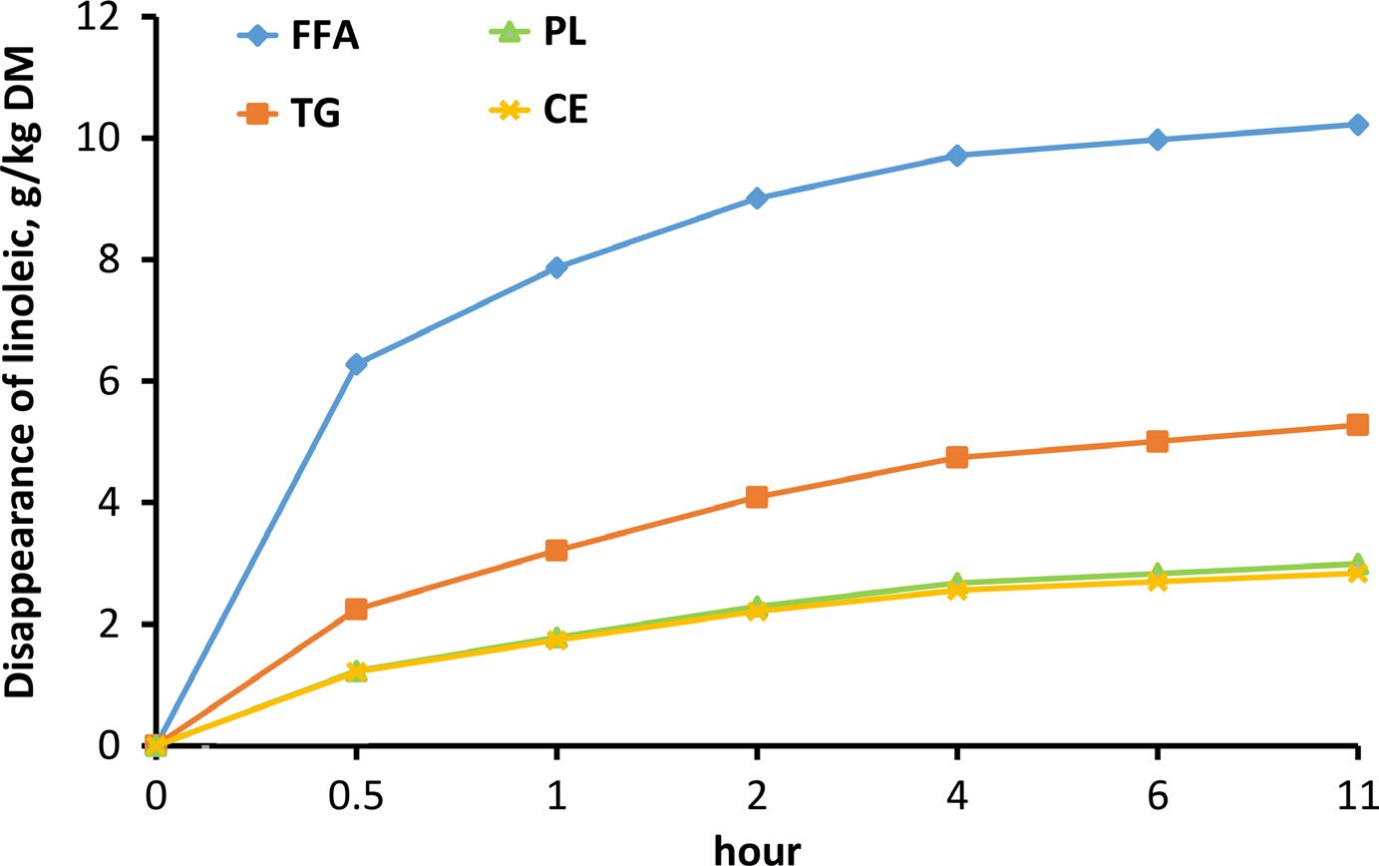
Disappearance of linoleic acid as free fatty acid (FFA), esterified to phospholipids (PL), triglycerides (TG) or cholesterol esters (CE) modeled according to Michaelis–Menten equation

Validation of in vitro procedure has been tested and published by Lashkari et al. (2017). The Hanes plot was used to describe the disappearance of LNA and LA and the appearance of SA and showed correlation coefficients between 0.92 and 0.99. The obtained curves fitted nicely to the Michaelis–Menten equation (Figures 1 and 2).

In general, the amount of BH in LNA and LA modeled according to Michaelis–Menten occurred in the following order: FFA > TG > PL, regardless of the FA esterified to the different lipid fractions. The V_max_ value in FFA_LNA and TG_LNA was higher than the PL_LNA, while the K_M_ value in PL_LNA tended to be greater than the FFA_LNA and TG_LNA (Table 2). These differences are highlighted in Figure 1, where BH of LNA modeled according to Michaelis–Menten equation shows the lower BH when LNA is esterified to PL compared with both FFA and TG.

**Table 2:** V_max_ (g FA/kg DM) and K_M_ (hour) values for in vitro disappearance of linolenic acid in different lipid fractions

	Lipid fractions			*SEM*	*p‐*Value
	FFA	TG	PL§		
V_max_ †	10.8^a^	9.3^a^	0.55^b^	0.36	<.001
K_M_ ‡	0.67	0.45	1.15	0.19	.10

Note

^ab^ Means within a row with different superscripts differ significantly (*p* < .05).

Abbreviations: FFA, free fatty acid; PL, phospholipids; *SEM*, standard error of means; TG, triglycerides.

^†^ V_max_: the maximum amount of fatty acid biohydrogenation (g/kg DM**)**.

^‡^ K_M_: the time need to achieve 50% of asymptotic maximum amount of fatty acid biohydrogenation (hours).

^§^ Phospholipids contain both linoleic and linolenic acids.

The BH of LA modeled according to Michaelis–Menten followed the same trend as seen for LNA in FFA, TG, and PL (Figure 2). The V_max_ of FFA_LA was significantly higher than that of TG_LA, PL_LA, and CE_LA (*p* < .05, Table 3). There was no significant difference in K_M_ and V_max_ values for SA formation between different lipid fractions (Table 4).

**Table 3:** V_max_ (g FA/kg DM) and K_M_ (hour) values for in vitro disappearance of linoleic acid in different lipid fractions

	Lipid fractions				*SEM*	*p‐*Value
	FFA	TG	CE	PL§		
V_max_ ^†^	10.5^a^	5.64^b^	3.03^b^	3.21^b^	0.78	<.001
K_M_ ^‡^	0.34	0.76	0.75	0.80	0.29	.66

Note

^ab^Means within a row with different superscripts differ significantly (*p* < .05).

Abbreviations: CE, cholesterol esters; FFA, free fatty acid; PL, phospholipids; *SEM*, standard error of means; TG, triglycerides.

^†^ V_max_: the maximum amount of fatty acid biohydrogenation (g/kg DM**)**.

^‡^ K_M_: the time need to achieve 50% of asymptotic maximum amount of fatty acid biohydrogenation (hours).

^§^ Phospholipids contain both linoleic and linolenic acids.

**Table 4:** V_max_ (g FA/kg DM) and K_M_ (hour) values for in vitro formation of stearic acid in different lipid fractions

	Lipid fractions						*SEM*	*p‐*Value
	FFA_LNA	TG_LNA	FFA_LA	TG_LA	CE_LA	PL§		
V_max_ ^†^	7.67	10.10	7.25	12.47	8.33	8.99	1.45	1.45
K_M_ ^‡^	1.79	6.01	2.94	1.99	1.37	4.00	0.20	.28

Note

Mean values were not different.

Abbreviations: CE, cholesterol esters; FFA, free fatty acid; LA, linoleic acid; LNA, linolenic acid; PL, phospholipids; *SEM*, standard error of means; TG, triglycerides.

^†^ V_max_: the maximum amount of stearic acid formation (g/kg DM)

^‡^ K_M_: the time need to achieve 50% of asymptotic maximum amount of stearic acid formation (hours)

^§^ Phospholipids contain both linoleic and linolenic acids.

### 3.3 Formation of conjugated linoleic acid isomers in different lipid fractions during the in vitro incubation

In the present study, the estimation of appearance rate for CLA isomers with the Michaelis–Menten is not reliable and accurate due to a small CLA pool size, and the fact that CLA isomers are intermediates. However, the BH pattern of cis‐9, trans‐11 CLA and trans‐10, cis‐12 CLA was affected by lipid fractions as shown in Table 5. The highest amount of cis‐9, trans‐11 CLA was found in FFA_LA after 0.5, 1, 2, and 4 hr of incubation, while after 6 and 11 hr of incubation, the amount of cis‐9, trans‐11 CLA was the highest in both FFA_LA and TG_LA (Table 5). The highest trans‐10, cis‐12 CLA amount after 0.5 and 1 hr of incubation was observed in FFA_LNA, while after 11 hr of incubation, the highest amount was observed in TG_LNA and FFA_LA.

**Table 5:** Amount of cis‐9, trans‐11 conjugated linoleic acid (CLA) and trans‐10, cis‐12 CLA during the in vitro incubation in different lipid fractions (mg/kg DM)

Time		Lipid fractions						*SEM*	*p‐*Value
		FFA_LNA	TG_LNA	FFA_LA	TG_LA	CE_LA	PL§		
0.5 hr after incubation	Cis‐9, trans‐11 CLA	4.9^cY^	3.4^c^	56.2^aX^	17.0^bX^	5.7^cX^	23.2^bX^	10.2	.004
	Trans‐10, cis‐12 CLA	15.9^aX^	3.4^b^	1.7^bY^	2.0^bY^	2.2^bY^	4.4^bY^	3.2	<.0001
	*SEM*	2.8	0.2	13.1	5.1	0.8	4.7	–	–
	*p‐*Value	.001	.10	<.0001	<.0001	.001	<.0001	–	–
1 hr after incubation	Cis‐9, trans‐11 CLA	4.8^cY^	3.1^cY^	52.8^aX^	18.8^bX^	4.2^cX^	16.2^bX^	10.3	<.0001
	Trans‐10, cis‐12 CLA	21.9^aX^	4.0^bX^	1.9^bY^	2.9^bY^	2.0^bY^	3.0^bY^	4.4	<.0001
	*SEM*	4.1	0.4	11.5	3.7	0.5	3.0	–	–
	*p‐*Value	<.0001	.04	<.0001	.001	.01	.005	–	–
2 hr after incubation	Cis‐9, trans‐11 CLA	3.1^cY^	3.4^cY^	51.9^aX^	17.0^bX^	4.0^cX^	13.2^bX^	10.5	<.0001
	Trans‐10, cis‐12 CLA	16.6^aX^	4.9^bX^	17.8^aY^	2.9^bY^	1.9^bY^	2.0^bY^	4.3	.001
	*SEM*	3.4	0.4	8.4	3.5	0.5	2.5	–	–
	*p‐*Value	<.0001	.001	<.0001	<.0001	.0001	.001	–	–
4 hr after incubation	Cis‐9, trans‐11 CLA	2.7^cY^	2.2^cY^	35.2^aX^	18.1^bX^	2.0^c^	11.4^bX^	7.3	<.0001
	Trans‐10, cis‐12 CLA	19.9^aX^	7.2^bX^	19.9^aY^	7.2^bY^	1.9^c^	2.6^cY^	3.7	.04
	*SEM*	2.6	1.2	3.9	2.7	0.3	2.4	–	–
	*p‐*Value	<.0001	.03	.001	<.0001	.15	.001	–	–
6 hr after incubation	Cis‐9, trans‐11 CLA	3.7^bY^	2.7^bY^	34.5^aX^	23.5^aX^	2.8^b^	9.1^cX^	7.5	.0002
	Trans‐10, cis‐12 CLA	14.8^bX^	9.7^bX^	23.1^aY^	11.2^bY^	2.2^c^	4.8^cY^	4.3	.008
	*SEM*	2.5	1.6	3.2	3.9	0.2	1.1	–	–
	*p‐*Value	<.0001	.001	.005	<.0001	.25	.02	–	–
11 hr after incubation	Cis‐9, trans‐11 CLA	3.4^bY^	3.8^bY^	30.4^aX^	25.2^aX^	4.3^b^	9.7^bX^	6.6	.008
	Trans‐10, cis‐12 CLA	13.9^bX^	16.0^aX^	18.5^aY^	14.1^bY^	6.9^c^	4.6^cY^	3.5	<.0001
	*SEM*	2.4	2.8	3.1	3.2	1.3	1.6	–	–
	*p‐*Value	<.0001	<.0001	.005	.008	.15	.009	–	–

Note

^abc^ Means within a row with different letters indicate significant differences (*p* < .05). ^XY^ Means within a column with different letters indicate significant differences (*p* < .05).

Abbreviation: CE, cholesterol esters; FFA, free fatty acid; LA, linoleic acid; LNA, Linolenic acid; PL, phospholipids; *SEM*, standard error of means; TG, triglycerides.

^§^ Phospholipids contain both linoleic and linolenic acids.

Comparison of amount of CLA isomers in same lipid fraction showed that irrespective of incubation time, the amount of cis‐9, trans‐11 CLA in FFA_LA, TG_LA, and LP was higher than the amount of trans‐10, cis‐12 CLA, while in CE_LA, no significant difference was observed between the amount of cis‐9, trans‐11 and trans‐10, cis‐12 CLA after 4, 6, and 11 hr of incubation. In contrast, the trans‐10, cis‐12 CLA amount in FFA_LNA and TG_LNA was higher than the cis‐9, trans‐11 CLA amount, regardless of incubation time.

## 4 DISCUSSION

### 4.1 Fatty acid methyl ester procedure

Various methods are available for rumen FA analyses, but no single method solves all the difficulties posed by the FA analysis. Difficulty may occur in quantitatively preparing FAME from such a great variety of FA and lipid classes that are present in rumen content. To overcome the difficulties in the preparation of FAME, due to heterogeneity of rumen FA, the combination of basic and acidic method has been highly recommended to methylate all lipid classes including triglycerides, phospholipids, sphingomyelins, steroid esters, and FFA (Kramer et al., 1997; Lashkari & Jensen, 2017). Some FAME preparation procedures are not able to methylate FFA (Kramer et al., 1997; Lashkari & Jensen, 2017), and in such cases, acidic methods are required, despite the risk of CLA isomerization (Kramer et al., 1997; Lashkari & Jensen, 2017).

### 4.2 Dose independency of Michaelis–Menten kinetics

In order to study BH kinetics, Michaelis–Menten kinetics has been used in previous studies (Lashkari et al., 2017). In the present study, rumen BH modeled according to Michaelis–Menten equation showed high R‐square (*R* ^2^ = 0.98 for CE_LA as an example). Michaelis–Menten kinetics is well known as a first‐order kinetic model to describe the enzymatic kinetics (Berg, Tymoczko, & Stryer, 2002). The kinetic constants (K_M_ and V_max_) are dose‐independent (Mehvar, 2001) and are therefore very useful to study the kinetic parameters in samples containing different concentrations of substrate such as PUFA. Other models have shown that BH kinetics depends on the concentration of unsaturated FA, and Beam, Jenkins, Moate, Kohn, and Palmquist (2000) reported when soybean oil level increased from 0% to 6% in in vitro condition, the BH rate of LA decreased from around 12%/hr to around 9%, and overall rate of LA BH decreased 1.2%/hr for each percent increase in LA added to substrate.

### 4.3 Biohydrogenation of LNA and LA esterified to different lipid fractions

The highest V_max_ value of the FFA in both LNA and LA was expected, as the exposure of the carboxyl group is a prerequisite to take place the BH. No significant difference for V_max_ value in TG_LNA and FFA_LNA demonstrated the high and rapid hydrolyze of TG_LNA as shown previously (Garton, Hobson, & Lough, 1958). The high lipolysis rate would be an explanation for the higher LNA and LA BH in TG than the other esterified forms. The results of the present study showed a lower V_max_ for LNA and LA when esterified to PL compared with FFA_LNA and FFA_LA. In agreement with our findings, a negative correlation between BH of LNA and the content of membrane lipids has been reported in common vetch (*Vicia sativa*) and crimson clover (*Trifolium incarnatum*) (Cabiddu et al., 2010). The membrane lipids included both galactolipids and PL show some inhibitory effects on BH of LNA (Cabiddu et al., 2010), maybe due to reduced lipolysis. The influence of lipid fractions on lipolysis and BH is proved (Chow et al., 2004), and this may be related to less availability of esterified FA for rumen microbes (Min, Barry, Attwood, & McNabb, 2003).

The high V_max_ value in both FFA_LNA and FFA_LA may be due to reduced adverse effects of free PUFA (Lashkari et al., 2017). It is well established that free PUFA are more toxic than esterified PUFA to the function of rumen bacteria; therefore, the microbes biohydrogenate PUFA to avoid the toxic effect (Choi et al., 2009). In the present study, the V_max_ and K_M_ values of SA formation in different lipid fractions were unchanged, indicating that neither the decrease in substrate concentrations nor the accumulation of BH intermediates altered the SA formation (Chowdhury, Lashkari, Jensen, Ambye Jensen, & Weisbjerg, 2018).

The high V_max_ value in FFA_LNA and FFA_LA implies that rumen BH of LNA and LA was higher in FFA compared with the PL. Previous studies in sheep have shown the similar differences between BH of free and esterified FA (Dawson & Hemington, 1974). The significant difference between cis‐9, trans‐11 and trans‐10, cis‐12 CLA amount in TG_LA and PL demonstrated the obvious effects of lipid fractions on isomerization process, preceding the first BH step. These findings show the importance of lipid fractions on lipolysis and BH as previously shown with fish oil (Chow et al., 2004).

The BH of LA (Figure 2) followed the same trends as for LNA (Figure 1), with no significant effect between BH of LA in PL and CE. This was unexpected, as rumen content of the plant sterols (Dawson & Hemington, 1974) and esterified tocopherols (Hymøller & Jensen, 2010) have been reported to remain fairly stable against rumen lipolysis over time, while rumen content of PL has been reported to decline in the rumen over time. Regardless of the individual PL investigated, the activity of phospholipase has been shown to be high in the rumen (Dawson, 1959), while the activity of the esterase enzymes seems to be highly dependent on the FA esterified to the sterol investigated (Hespell & O'Bryan‐Shah, 1988; Lanz & Williams, 1973). Hymøller and Jensen (2010) showed that α‐tocopheryl acetate is not hydrolyzed in the rumen of dairy cows; therefore, the lowest BH in CE might be due to the low esterase activity. The missing differences in BH of LA between the PL and CE fractions could be concealed by the approximately 2% of free LA in the CE, which is biohydrogenated fast, and this may consequently cause an overestimation of the K_M_ and V_max_ values in the present study. The literature also seems consistent in the low esterase activity in the rumen, regardless of the sterol ester source, and it has been reported that the activity of the esterase enzymes was reduced when either 1‐naphthyl acetate (Lanz & Williams, 1973) was the source of sterol esters in in vitro condition or α‐tocopheryl acetate (Hymøller & Jensen, 2011) was the source of steroid in in vivo study.

The lower V_max_ in PL_LNA (0.55 g FA/kg DM) compared with PL_LA (3.21 g FA/kg DM) may arise from competition between LNA and LA for the initial step of BH, already hypothesized by Troegeler‐Meynadier, Nicot, Bayourthe, Moncoulon, and Enjalbert (2003). The isomerization of the cis‐12 double bond into trans‐11 is a key step before saturation (Chow et al., 2004) and is probably performed on the same site for LA and LNA (Kepler & Tove, 1967). Most likely, LNA and LA compete for the sites, causing a lower BH of LNA in the PL incubations at their start, without affecting the global BH.

### 4.4 Formation of conjugated linoleic acid isomers in different lipid fractions during the in vitro incubation

The results showed that the FFA_LNA and TG_LNA produced more trans‐10, cis‐12 CLA than cis‐9, trans‐11 CLA compared with the corresponding lipid fractions containing the LA, as they produced more cis‐9, trans‐11 CLA than the trans‐10, cis‐12 CLA. These results show that LNA may change the rumen BH in favor of trans‐10, cis‐12 CLA formation. In agreement with our findings, the high amount of trans‐10, cis‐12 CLA reported in duodenal contents from cattle fed diet with the high amount of LNA (Loor, Bandara, & Herbein, 2002). These results may show that the enzyme‐catalyzed reactions leading to the formation of CLA isomers response differently to the different FAs. Also, the geometry of FAs binding to the isomerase enzyme could be a reason for differences in cis‐9, trans‐11 CLA and trans‐10, cis‐12 CLA amount between LNA and LA as previously reported by Liavonchanka, Hornung, Feussner, and Rudolph (2006). These findings showed that the level of understanding of the mechanisms of enzyme‐catalyzed reactions leading to the formation of CLA isomers differs across the lipid fractions and specific PUFA. In addition, different amounts of CLA isomers formed in different lipid fractions and in the same lipid fraction containing different FAs might reflect that the formation of CLA isomers may occur via a different biochemical mechanism. However, the results of CLA formation should be interpreted with caution, because of the slight difference in the concentration of LA and LNA.

## 5 CONCLUSION

In conclusion, the present in vitro study suggests that PUFA in complex esters are more resistant to in vitro BH, which ultimately results in decreased BH of PUFA when esterified to PL or CE fractions. In addition, the results of the present study show that the LNA may change the rumen BH in favor of trans‐10, cis‐12 CLA formation, regardless of lipid fractions.

## CONFLICT OF INTEREST

The authors declare no conflict of interest.

## ETHICAL APPROVAL

The experiment was carried out at Aarhus University, Department of Animal Sciences, Denmark, and was carried out in accordance with the Danish Ministry of Justice Law No. 1306 (23 November 2007) regarding animal experiments and care of experimental animals.
